# Viruses and bacteria in floodplain lakes along a major Amazon tributary respond to distance to the Amazon River

**DOI:** 10.3389/fmicb.2015.00158

**Published:** 2015-03-04

**Authors:** Rafael M. Almeida, Fábio Roland, Simone J. Cardoso, Vinícius F. Farjalla, Reinaldo L. Bozelli, Nathan O. Barros

**Affiliations:** ^1^Laboratory of Aquatic Ecology, Department of Biology, Federal University of Juiz de Fora, Juiz de ForaBrazil; ^2^Department of Sanitary and Environmental Engineer, Federal University of Juiz de Fora, Juiz de ForaBrazil; ^3^Laboratory of Limnology, Federal University of Rio de Janeiro, Rio de JaneiroBrazil

**Keywords:** plankton, viruses, bacteria, Amazonian freshwater ecosystems, floodplain lakes, dissolved organic carbon, backwater effect

## Abstract

In response to the massive volume of water along the Amazon River, the Amazon tributaries have their water backed up by 100s of kilometers upstream their mouth. This backwater effect is part of the complex hydrodynamics of Amazonian surface waters, which in turn drives the variation in concentrations of organic matter and nutrients, and also regulates planktonic communities such as viruses and bacteria. Viruses and bacteria are commonly tightly coupled to each other, and their ecological role in aquatic food webs has been increasingly recognized. Here, we surveyed viral and bacterial abundances (BAs) in 26 floodplain lakes along the Trombetas River, the largest clear-water tributary of the Amazon River’s north margin. We correlated viral and BAs with temperature, pH, dissolved inorganic carbon, dissolved organic carbon (DOC), phosphorus, nitrogen, turbidity, water transparency, partial pressure of carbon dioxide (pCO_2_), phytoplankton abundance, and distance from the lake mouth until the confluence of the Trombetas with the Amazon River. We hypothesized that both bacterial and viral abundances (VAs) would change along a latitudinal gradient, as the backwater effect becomes more intense with increased proximity to the Amazon River; different flood duration and intensity among lakes and waters with contrasting sources would cause spatial variation. Our measurements were performed during the low water period, when floodplain lakes are in their most lake-like conditions. Viral and BAs, DOC, pCO_2_, and water transparency increased as distance to the Amazon River increased. Most viruses were bacteriophages, as viruses were strongly linked to bacteria, but not to phytoplankton. We suggest that BAs increase in response to DOC quantity and possibly quality, consequently leading to increased VAs. Our results highlight that hydrodynamics plays a key role in the regulation of planktonic viral and bacterial communities in Amazonian floodplain lakes.

## INTRODUCTION

Of the 10 largest tropical rivers in terms of discharge on Earth, four are in the Amazon basin, being the Amazon River itself the largest one (Latrubesse et al., 2005). In addition to a complexly arranged fluvial network, the Amazon floodplain is composed of extensive wetlands and about 9,000 floodplain lakes that cover nearly 70,000 km^2^ (McClain, 2001), which are seasonally flooded by bordering rivers. The flood pulse is one of the most marked characteristics of Amazonian aquatic ecosystems (Junk et al., 1989), and it defines four distinct flood seasons: rising, high, falling, and low waters. Because peak discharges of the northern and southern tributaries of the Amazon River have different timings, the discharges of the Amazon River vary by a factor of 3, whereas its tributaries vary their discharges by a factor of 10 (Meade et al., 1991). As a result, even the largest tributaries have their water backed up by 100s of kilometers upstream of the mouth, with falling river stages being as much as 3 m higher than rising stages at a same discharge – the so-called backwater effect. The intricate hydrodynamics of Amazonian aquatic systems regulates the concentrations of organic matter and nutrients in Amazonian lakes (Forsberg et al., 1988), as well as a variety of aquatic communities, such as the planktonic ones, zoo-, phyto-, bacterio-, and virioplankton (Bozelli, 1994; Anesio et al., 1997; Huszar and Reynolds, 1997; Barros et al., 2010).

Viruses are ubiquitous in aquatic ecosystems, and increasing attention has been paid on their role in aquatic food webs since it was discovered that they are the most abundant aquatic components (Bergh et al., 1989). Viruses are not only abundant, but they also play an important biogeochemical function by releasing dissolved organic matter (DOM) and nutrients through host cell lysis (Fuhrman, 1999). In addition, viral activity can affect ecosystem respiration, primary production, bacterial and algal diversity, species distribution, and genetic transfer between microorganisms (Maranger and Bird, 1995; Fuhrman, 1999; Suttle, 2005). Likewise, bacteria are crucial players in aquatic ecosystems, processing large amounts of both autochthonously and allochthonously derived organic carbon (Cotner and Biddanda, 2002). For this reason, bacteria and viruses are recognized as key alternative routes of organic matter and nutrient transfer to metazoan trophic levels, which was first introduced through the microbial loop concept (Pomeroy, 1974; Azam et al., 1983), and then by the viral loop (Fuhrman, 1999).

While temperature, nutrient, organic carbon, flood pulses, and light exposure are key bottom–up factors controlling bacterial dynamics in aquatic systems (Farjalla et al., 2002, 2006; Amado et al., 2013), the action of viruses is known to be an important top–down mechanism of bacterial regulation in aquatic ecosystems (Fuhrman and Noble, 1995). This viral control on bacteria is summarized in the “killing the winner” hypothesis: abundant prokaryotic types are exposed to strong viral pressure, because viral infection rate depends, among other things, on the abundance and type of prokaryotic host cells (Winter et al., 2010). Viruses impact directly on bacterial populations and indirectly on bacterial diversity by decreasing the density of dominant bacterial species (Maranger and Bird, 1995). Moreover, viruses can account for up to 40% of bacterial mortality in surface waters, which can be similar in magnitude to the effect caused by protistan grazing (Fuhrman and Noble, 1995).

Although there is a growing body of research on aquatic viral ecology, little is known about viral function in tropical environments (Peduzzi and Schiemer, 2004; Bettarel et al., 2006; Araújo and Godinho, 2009). This is particularly true for the Amazon, where to our knowledge only one study has investigated aquatic viruses to date (Barros et al., 2010). In Amazonian clear-water floodplain lakes, bacterial and viral abundances (VAs) are tightly coupled, and both of them are linked to the flood pulse and the concentration of suspended particles (Barros et al., 2010). This study was a key initial step toward a comprehensive understanding on the role of viruses in Amazonian aquatic ecosystems, but the variation of bacteria and virus between contrasting floodplain lakes is still unknown. Spatially explicit reports of virus–bacterium relationships have been documented for boreal, temperate and tropical African lakes (Maranger and Bird, 1995; Anesio et al., 2004; Bettarel et al., 2006), but no such study exists for the Neotropical region.

Here, we made an extensive survey of several floodplain lakes distributed along the margins of the Trombetas River, the second largest northern tributary of the Amazon River. Throughout the sampling stretch, the Trombetas River is permanently subject to a backwater effect caused by the Amazon River (Veiga Pires et al., 1988). The backwater effect becomes progressively more pronounced with increasing proximity to the confluence with the Amazon River (Meade et al., 1991). As a result, lower basin lakes are frequently flooded by the turbid waters of the Amazon River, whereas upper basin lakes are strictly flooded by the clear waters of the Trombetas River. In addition, the duration of floods may be longer in the lower basin. Thus, we hypothesized that both bacterial and VAs would change according to the distance to the Amazon River. This would occur because of different flood duration and intensity among lakes and waters with different sources. Contrasting flooding characteristics would eventually influence planktonic communities during low water, when lakes are more disconnected and dissimilar (Thomaz et al., 2007).

## MATERIALS AND METHODS

### SITE DESCRIPTION

The Trombetas River originates in the Guiana shield and is the largest northern clear-water tributary of the Amazon River, with a mean discharge of 2,555 m^3^ s^-1^ (Moreira-Turcq et al., 2003). The total area of the Trombetas River basin is 120,000 km^2^, 6% of which are covered by floodplain forests and lakes (Melack and Hess, 2010). These floodplain lakes exhibit large oscillation in water level over the year, with mean depths being as low as 1 m during low water periods and as high as 10 m during high water periods (Roland and Esteves, 1998).

**FIGURE 1:**
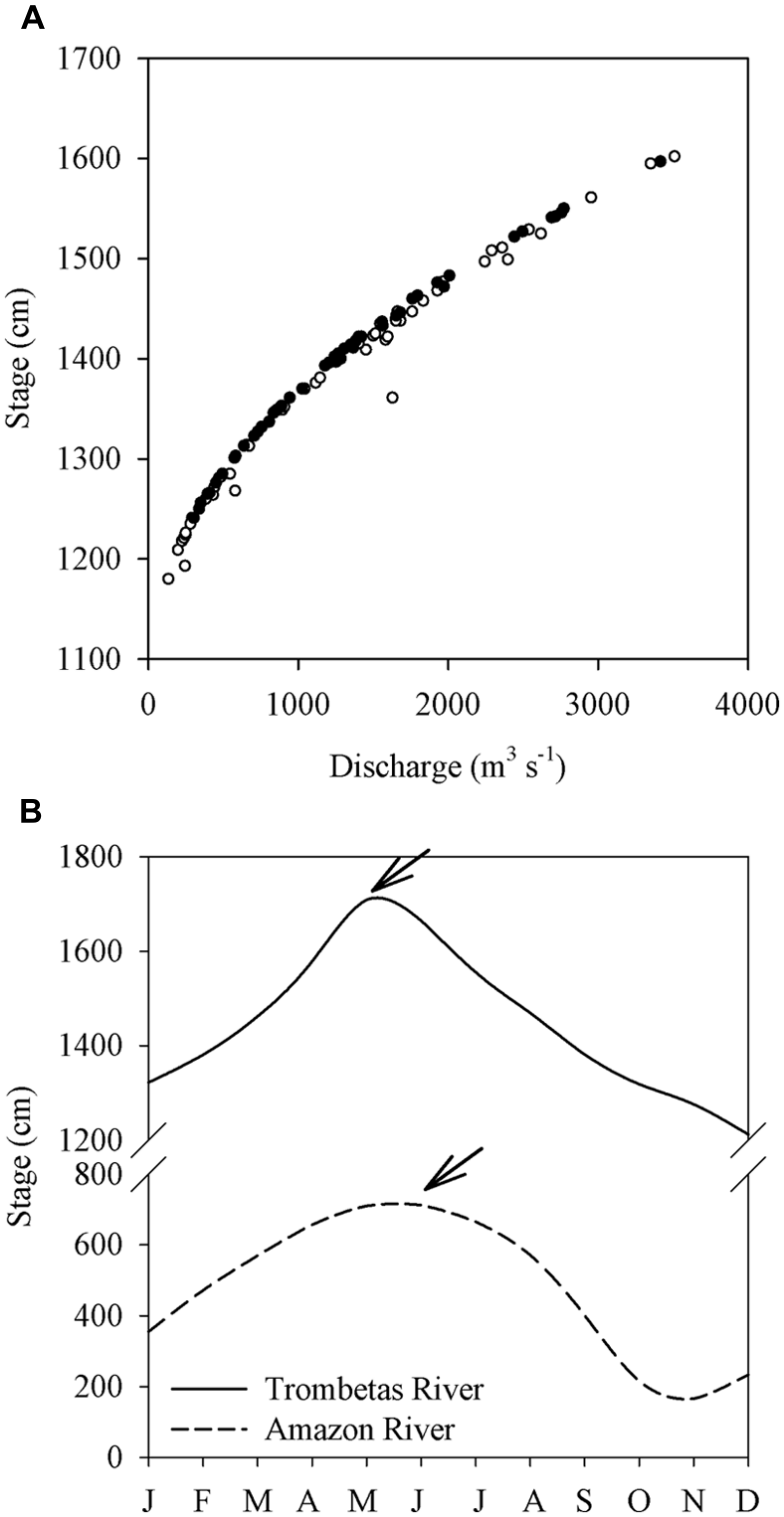
**(A)** Stage-discharge relations in the Trombetas River at the Caramujo gaging station (1∘3′54^″^S, 57∘3′41^″^W) during the rising (open circles) and falling (solid circles) stages. **(B)** Mean stages of the Trombetas River at Caramujo between 1996 and 2013 (solid line) and the Amazon River at Óbidos (1∘ 55′9^″^S, 55∘30′47^″^W) between 1968 and 2013 (dashed line); the X-axis indicates the months of the year. The arrows indicate the peak discharges of both rivers. Data were obtained downstream the confluence of both rivers. Discharges and stages data were obtained at the website of the Brazilian National Water Agency (http://hidroweb.ana.gov.br).

The backwater effect of the Amazon River on its tributaries is a pattern well described in literature, and it gets gradually more pronounced following an upstream–downstream gradient (Meade et al., 1991). The Trombetas River, for instance, has been reported to be permanently subject to a backwater effect until Cachoeira Porteira, about 210 km upstream the mouth (Veiga Pires et al., 1988). Discharge and stage measurements from a gaging station 20 km upriver of our upper-most lake (Lake Macaco) confirm this (**Figure 1A**). At this gaging station, the river level is sometimes a few centimeters higher during falling stages than during rising stages at the same discharge, which is due to a time lag between the peak discharges of the Trombetas and Amazon rivers (**Figure 1B**).

**FIGURE 2:**
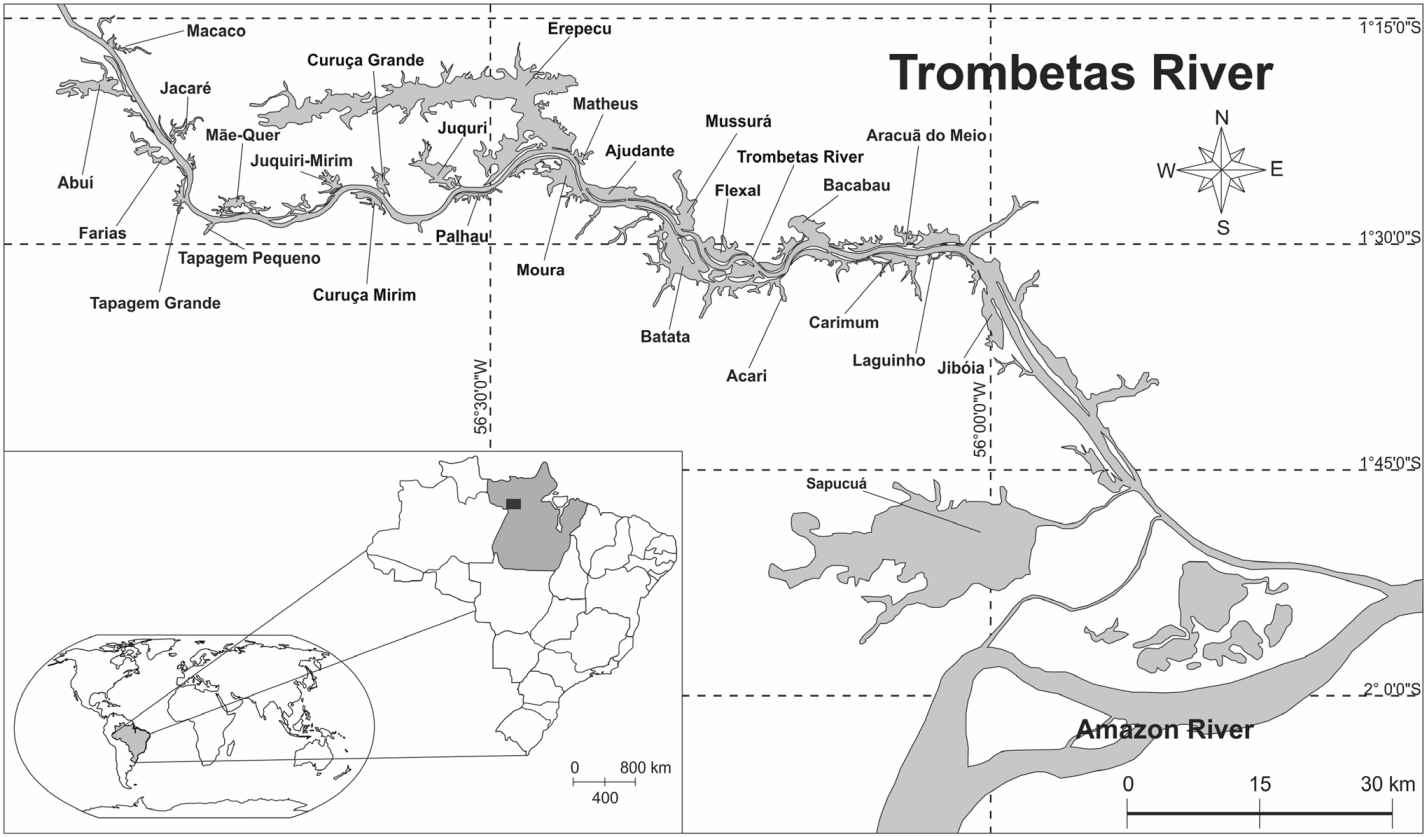
**Map of the study site, emphasizing the Trombetas River and its associated floodplain lakes**.

We selected the peak of a low water period to perform our measurements, in order to sample the floodplain lakes in their most lake-like conditions. During low water, river-floodplain systems are more heterogeneous with respect to physical, chemical and biological variables, since the connectivity with the adjoining river is weakest (Thomaz et al., 2007). We sampled 26 floodplain lakes adjacent to the Trombetas River, following a north (upstream) to south (downstream) gradient (**Figure 2**). The northern-most lake is about 200-km distant from the confluence of the Trombetas with the Amazon River. We also sampled one site in the Trombetas River, located halfway from the confluence with the Amazon River until the northern-most lake. Satellite imagery from the free software Google Earth was used to measure the distance traveled through the Trombetas River main channel from the lake mouths until the confluence with the Amazon River.

### WATER SAMPLING AND ANALYSIS

Water samples were taken from the upper 0.5 m at the center of each of the 26 lakes during a cruise along the Trombetas River between December 1st and 8th 2007 (low water period). In the field, subsamples were filtered through 0.7 μm glass microfiber filters (GF/F; Whatman) for further analysis of dissolved compounds. All water samples sent for laboratory analysis were kept refrigerated at ∼4∘C and analyzed within 15 days after sampling. Dissolved oxygen (DO) concentrations and water temperature were measured at the subsurface with a portable oximeter (YSI-95) and a thermometer, respectively. Turbidity was measured using a turbidimeter La Motte 2008 Turbidity Meter, whereas samples for pH were analyzed using a pH meter (Micronal B474). The water transparency was measured using the Secchi disk. Pre-acidified (pH < 2) water samples for total nitrogen (TN) and total phosphorus (TP) were analyzed within 15 days using standard spectrophotometric techniques (Wetzel and Likens, 2000). Pre filtered samples of DOC and dissolved inorganic carbon (DIC) were analyzed on a Tekmar–Dohrmann Total Carbon Analyzer (model Phoenix 8000). DIC was analyzed following persulfate digestion, and pre-acidified (pH < 2) DOC samples were analyzed following high temperature oxidation with a UV lamp. The partial pressure of carbon dioxide (pCO_2_) was calculated from DIC, pH, and water temperature according to Stumm and Morgan (1996).

### VIRAL, BACTERIAL, AND PHYTOPLANKTON ABUNDANCES

Here, we consider bacteria as a generic term describing prokaryotic organisms (i.e., organisms lacking a nucleus, comprising the domains *Bacteria* and *Archaea*), since the method that we used (epifluorescence microscopy using SYBR stains) does not distinguish bacteria from Archaea. Additionally, free DNA and non-viral background fractions occasionally interferes the counting of viruses through epifluorescence miscroscopy using SYBR stains (Pollard, 2012). Samples for viral and bacterial abundances (BAs) were taken from the center of the lakes in triplicates. Immediately after sampling, the samples were fixed with glutaraldehyde solution (2% final concentration; pre-filtered on a 0.02 μM-pore-size filter). In the laboratory, on the same day of sampling, bacteria and viruses were stained with SYBR green (Molecular Probes, Eugene, OR, USA; Noble and Fuhrman, 1998), which is recommendable as immediate preparation of slides avoids viral decay that commonly occurs during storage of water samples. Two-milliliter samples were filtered on a 0.02 μm-pore-size Anodisc membrane filter (Whatman aluminum oxide) with a 0.45 μm-pore-size backing membrane filter. The filter was laid, sample side up, on a drop of SYBR green I solution (1:400) for 15 min in the dark. After being dried, the filter was placed on a glass slide and mounted with an antifade mounting solution (Patel et al., 2007), and kept frozen at -20∘C until analysis within 15 days after sampling. For each filter, more than 200 viruses and 100 bacteria were directly counted in 20 fields. The fields were selected randomly. Analyses were performed under ×1,000 magnification with an epifluorescence microscope (Provis AX-70; Olympus, Melville, NY, USA) using light filters for blue excitation (450–490 nm wide bandpass).

Samples for phytoplankton enumeration were fixed in the field with acidic Lugol’s solution at a final concentration of 1:100 (Soares et al., 2011). The samples were stored in dark glass-ware protected from light and analyzed within 15 days after sampling. In the laboratory phytoplankton abundances (PAs) were determined in an inverted microscope (Olympus IX 71) following the Utermöhl (1958) sedimentation method. At least one 100 specimens of the dominant species were enumerated (Lund et al., 1958) in random fields (Uhelinger, 1964).

### STATISTICAL ANALYSIS

We used linear regressions to assess possible relationships between distance to the Amazon River and the limnological parameters and planktonic communities considered here. A linear regression was also used to verify the coupling between bacteria and viruses. We utilized *p* < 0.05 as a threshold level for the acceptance. All analyses were made on SigmaPlot version 11.0.

## RESULTS

The distance from the lake mouths until the Amazon River varied between 31 km (Sapucuá Lake) and 192 km (Macaco Lake; **Table 1**). Water temperature was elevated and showed minor variation between lakes. Oxygen concentrations averaged 5.9 mg L^-1^ (range: 4.3–6.8 mg L^-1^), and were about 80% of the oxygen saturation considering the water temperature and atmospheric pressure. The apparent oxygen deficit in the water was corroborated by CO_2_ supersaturation (average pCO_2_ = 2916 μatm; range 320–5856 μatm). Only one lake was below atmospheric equilibrium, here considered as 390 μatm (**Table 1**). The Trombetas River, sampled 90 km upstream the mouth, showed oxygen concentrations and pCO_2_ similar to the average of the lakes, but it was less enriched in DOC, TN, and TP than most lakes. Turbidity was mostly below the detection limit in the upper basin lakes, as expected for clear-water systems. This is confirmed by the fact that the Secchi disk transparency (SDT) had a positive relationship with distance to the Amazon River (**Table 2**). The distance to the Amazon River was also significantly and positively correlated to DOC concentrations and pCO_2_ (**Table 2**).

**Table 1:** Limnological characteristics of the 26 lakes and the Trombetas River.

Lakes	Coordinates	Distance to Amazon River (km)	Depht (m)	Temp (∘C)	Secchi (m)	DO (mg L^-1^)	DOC (mg L^-1^)	DIC (mg L^-1^)	TP (μg L^-1^)	TN (μg L^-1^)	Turbitidy (NTU)	pH	pCO_2_ (μatm)
Sapucuá	S 1∘47′ 25^″^, W 55∘ 59′ 38^″^	31	2.2	28.8	0.4	5.5	4.6	2.7	87	577	31.8	7.7	320
Jibóia	S 1∘38′ 05^″^, W 55∘ 59′ 30^″^	54	1.7	30.5	1.3	6.7	3.4	1.0	26	240	1.4	6.3	1575
Laguinho	S 1∘31′ 43^″^, W 56∘ 04′ 07^″^	68	1.4	33.8	1.0	6.5	4.8	1.0	24	461	ND	5.0	2927
Aracu a do Meio	S 1∘ 30′ 48^″^, W 56∘ 07′ 37^″^	70	1.9	30.1	0.8	6.7	3.8	1.3	30	329	6.8	6.1	2323
Carimum	S 1∘ 31′ 37^″^, W 56∘ 06′ 06^″^	71	1.9	32.4	1.1	6.3	3.3	0.8	34	539	0.5	5.1	2312
Bacabau	S 1∘ 29′ 34^″^, W 56∘ 11′ 06^″^	82	1.9	30.5	1.2	5.7	4.6	1.5	28	363	0.7	5.5	3834
Acari	S 1∘ 33′ 06^″^, W 56∘ 13′ 11^″^	88	3.7	30.9	2.1	5.5	3.4	1.0	12	283	ND	5.1	2790
Flexal	S 1∘ 30′ 49^″^, W 56∘ 16′ 10^″^	95	2.4	30.7	0.9	5.4	4.3	1.1	31	503	4.6	5.3	2903
Batata	S 1∘ 31′ 56^″^, W 56∘ 18′ 31^″^	95	3.0	29.0	1.2	–	5.4	1.7	–	–	10.0	6.3	2430
Mussurá	S 1∘ 28′ 57^″^, W 56∘ 18′ 17^″^	100	2.4	30.1	0.5	5.5	3.9	1.8	27	469	18.6	6.4	2298
Ajudante	S 1∘ 27′ 21^″^, W 56∘ 22′ 45^″^	109	1.9	30.6	1.2	6.8	3.5	1.3	23	452	ND	5.9	2778
Moura	S 1∘ 25′ 36^″^, W 56∘ 25′ 4^″^	113	5.0	30.5	2.1	5.8	3.7	0.7	29	425	ND	5.4	1862
Matheus	S 1∘ 24′ 49^″^, W 56∘ 24′ 37^″^	115	1.6	32.6	1.6	6.5	5.6	0.8	30	568	ND	5.2	2373
Erepecu	S 1∘ 20′ 26^″^, W 56∘ 28′ 06^″^	119	4.2	31.5	1.3	6.1	4.3	1.0	27	426	ND	5.9	2249
Palhau	S 1∘ 26′ 46^″^, W 56∘ 31′ 14^″^	130	2.3	30.1	1.3	4.7	4.1	–	39	352	2.2	5.5	–
Juquiri-Grande	S 1∘ 25′ 02^″^, W 56∘ 34′ 06^″^	133	3.4	30.5	1.8	6.0	4.1	1.0	29	279	ND	5.4	2525
Curuçá-Mirim	S 1∘ 25′ 15^″^, W 56∘ 37′ 17^″^	145	2.9	31.7	1.9	5.9	4.2	1.1	37	504	ND	5.2	2943
Curuçá-Grande	S 1∘ 26′ 21^″^, W 56∘ 38′ 04^″^	145	2.1	32.4	1.7	6.0	5.5	1.1	24	510	ND	5.5	2908
Juquiri-Mirim	S 1∘ 24′ 55^″^, W 56∘ 40′ 13^″^	150	2.3	31.2	1.6	6.1	3.5	1.4	14	359	ND	5.2	3853
M ae-Quer	S 1∘ 25′ 55^″^, W 56∘ 46′ 58^″^	162	2.8	31.2	2.0	5.6	4.5	1.4	50	608	ND	5.5	3520
Tapagem Pequeno	S 1∘ 25′ 59^″^, W 56∘ 51′ 26^″^	170	2.0	29.9	1.4	5.6	4.4	1.4	16	304	ND	5.4	3487
Tapagem Grande	S 1∘ 24′ 36^″^, W 56∘ 51′ 13^″^	172	4.0	29.2	1.7	5.7	4.7	1.5	18	364	0.9	5.4	3817
Farias	S 1∘ 21′ 45^″^, W 56∘ 53′ 12^″^	178	1.5	32.6	1.3	6.6	7.0	1.5	30	660	ND	4.6	4438
Jacaré	S 1∘ 20′ 32^″^, W 56∘ 51′ 01^″^	178	2.1	32.7	1.0	6.8	5.4	1.3	41	577	0.4	5.3	3521
Abuí	S 1∘ 16′ 17^″^, W 56∘ 56′ 56^″^	184	2.5	32.4	1.7	6.2	4.0	0.9	18	417	ND	5.5	2457
Macaco	S 1∘ 12′ 51^″^, W 56∘ 53′ 50^″^	192	2.3	29.9	1.6	4.3	6.6	2.5	37	609	0.6	5.7	5856
Trombetas River	S 1∘ 31′ 22^″^, W 56∘ 14′ 46^″^	90	2.5	30.0	1.8	5.8	3.6	1.4	17	259	ND	5.3	3509

*Mean*	*–*	*–*	*2.5*	*31.0*	*1.4*	*5.9*	*4.5*	*1.3*	*30*	*440*	*3.0*	*5.6*	*2916*
*SD*	*–*	*–*	*0.9*	*1.3*	*0.5*	*0.6*	*1.0*	*0.5*	*15*	*121*	*7.1*	*0.6*	*1040*

*Temp., temperature; Secchi, Secchi disk transparency; DO, dissolved oxygen: DOC, dissolved organic carbon; DIC, dissolved inorganic carbon; TP, total phosphorus; TN, total nitrogen; pCO_2_, partial pressure of carbon dioxide.*

**Table 2:** Simple linear regression relationships.

REGRESSION EQUATION	*R*^2^	*p*-value	*n*
SDT = 0.751 + (0.00514*D)	*0.26*	*<0.05*	*26*
pCO_2_ = 903.0 + (16.470*D)	*0.50*	*<0.05*	*25*
logDOC = 0.537 + (0.000873*D)	*0.20*	*<0.05*	*26*
BA = 1.773 + (0.0205*D)	*0.24*	*<0.05*	*26*
VA = 1.051 + (0.00691*D)	*0.17*	*<0.05*	*26*
logVBR = 0.757 - (0.000801*D)	0.08	0.17	26
logPA = 3.186 + (0.000515*D)	0.01	0.79	26
*BA =* -1.315 + (8.668*logDOC)	*0.16*	*<0.05*	*26*
VA = -0.121 + (3.127*logDOC)	0.13	0.07	26
BA = 3.062 + (0.367*logPA)	0.01	0.69	26
VA = 2.598 - (0.219*logPA)	0.02	0.56	26
*BA =* -9.081 + (3.872*logpCO_2_)	*0.23*	*<0.05*	*26*
*VA =* -3.062 + (1.435*logpCO_2_)	*0.20*	*<0.05*	*26*

*D, distance traveled through the Trombetas River from the lake mouth until the Amazon River; SDT, Secchi disk transparency; pCO2, carbon dioxide partial pressure; DOC, dissolved organic carbon; BA, bacterial abundance; VA, viral abundance; VBR, virus-to-bacterium ratio; PA, phytoplankton abundance. The values were log transformed for DOC, VBR, and PA because the data failed the normality test (Shapiro–Wilk, *p* < 0.05). The significant relationships are shown in italics.*

**FIGURE 3:**
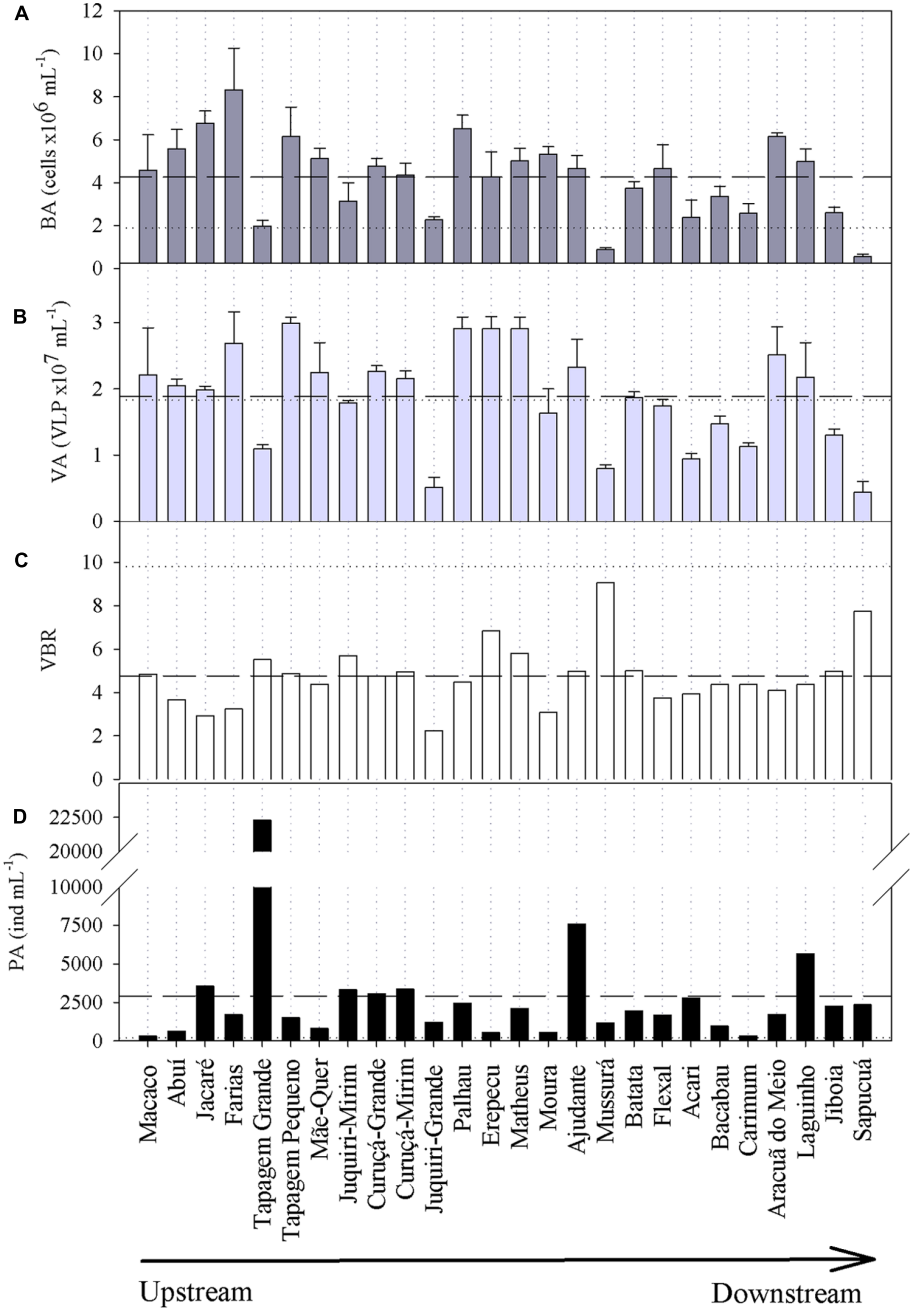
**(A)** Bacterial abundances (BA), **(B)** viral abundances (VA), **(C)** virus-to-bacterium ratios (VBR), and **(D)** phytoplankton abundances (PA) in the floodplain lakes of the Trombetas River basin. Bars and traces in **(A,B)** represent mean and SD of the bacterial and viral counting, respectively. Dashed and dotted horizontal lines indicate the average of the lakes and the value of the Trombetas River, respectively.

Viral abundances were higher than BAs irrespective of the sampling site (**Figure 3A**), averaging 1.9 × 10^7^ (±0.7 SD) VLP mL^-1^ (range: 0.4–3.0 × 10^7^ VLP mL^-1^); the VA of the Trombetas River (1.8 × 10^7^ VLP mL^-1^) was close to the average of the lakes. BAs varied by an order of magnitude, ranging from 0.6 × 10^6^ cells mL^-1^ to 8.3 × 10^6^ cells mL^-1^ (**Figure 3B**); the BA of the Trombetas River (1.9 × 10^6^ cells mL^1^) was in the lower range of the values found for the lakes. If on the one hand viral and BAs varied substantially between lakes, on the other hand, the virus-to-bacterium ratio (VBR) was less variable (**Figure 3C**), averaging 4.8 (±1.46 SD; range: 2.2–9.1), and it was higher in the Trombetas River (9.8) than in all lakes. The PAs varied considerably (range: 318–22300 ind. mL^-1^; **Figure 3D**), and the Trombetas River exhibited a very low PA (190 ind. mL^-1^).

**FIGURE 4:**
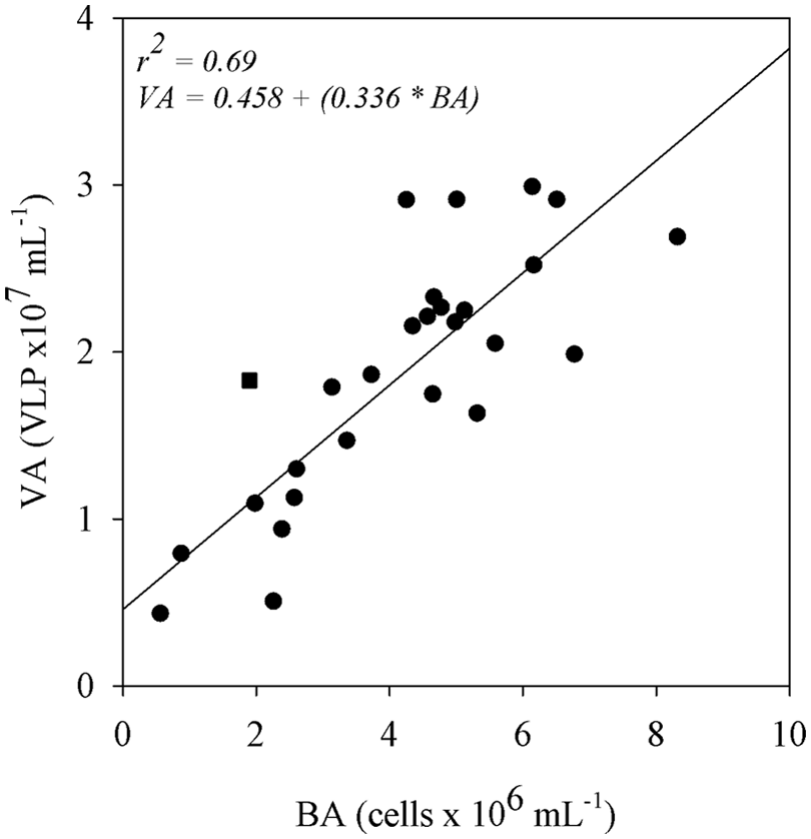
**Simple linear regression of BA with VA in the floodplain lakes.** The Trombetas River (square) was not included in the regression and is shown as outer data. The relationship was significant (*p* < 0.05).

A simple linear regression analysis showed that BA has a positive relationship with VA (*r*^2^ = 0.69; *p* < 0.05; **Figure 4**). On the other hand, no significant relationships were found when PAs were regressed against bacterial and VAs (**Table 2**). Both bacterial and VAs had a weak positive, but significant relationship with the distance from the lake mouths until the confluence of the Trombetas and Amazon rivers (*r*^2^ = 0.24; *p* < 0.05 and *r*^2^ = 0.17; *p* < 0.05, respectively; **Table 2**). DOC was significantly correlated to BAs (*r*^2^ = 0.16; *p* < 0.05), but not to VAs (**Table 2**). Both bacterial and VAs showed positive and significant relationship with pCO2 (*r*^2^ = 0.23; *p* < 0.05 and *r*^2^ = 0.20; *p* < 0.05, respectively; **Table 2**). The VBR was not significantly related to any variable (**Table 2**).

## DISCUSSION

### HYDRODYNAMICS AFFECTING WATER CHEMISTRY, BACTERIA, AND VIRUSES

The results of our analysis indicated that viral and BAs, DOC, pCO_2_, and water transparency of floodplain lakes adjacent to the Trombetas River increase as distance from the lake mouth until the Amazon River increases. We attribute this latitudinal gradient to a decreased intensity of the backwater effect of the Amazon River as one moves upriver. The intensity of the backwater effect in floodplain lakes bordering Amazon tributaries becomes progressively more pronounced with increasing proximity to the Amazon River (Meade et al., 1991). In other words, the backwater effect keeps the water level high during falling stages in lower basin lakes, which tend to present a higher proportion of river water with respect to local water at low water periods. The presence of riverine flood waters dilutes DOC, cells, and virus-like particles (Anesio et al., 1997; Farjalla et al., 2006; Barros et al., 2010), and increases allochthonous to autochthonous DOC ratio, as more terrestrially derived recalcitrant DOC enters the lakes (Farjalla et al., 2006). In addition, proximity to the Amazon River makes lakes subject to turbid flood waters, which is corroborated by the decreased SDT and increased turbidity in the lower basin lakes studied here. Also, we cannot discard the possibility that the higher turbidity in the lower basin lakes is partly anthropogenic, as this portion of the basin is closer to urban areas and human settlements. High turbidity implies that less labile autochthonous DOC is formed by primary producers. This is consistent with findings from Batata Lake, a clear-water floodplain lake heavily impacted by bauxite tailings where the turbid impacted sites exhibit lower DOC as well as bacterial and VAs than clear-water natural sites (Barros et al., 2010).

We suggest that a chain of events is triggered following the decreased intensity of the backwater effect as proximity to the Amazon River decreases: DOC increases (in quantity and likely in quality), leading to increased BAs, and ultimately VAs. It has been shown before that DOC stimulates bacterial growth, and that VAs respond to changes in BAs in a clear-water Amazonian lake (Farjalla et al., 2002; Barros et al., 2010). Finally, pCO_2_ also increased with distance to the Amazon River, which is probably a result of increased bacterial respiration due to higher BA. Indeed, BA was positively correlated to pCO_2_.

In addition to proximity to the Amazon River, it is likely that other factors also regulate bacteria and viruses in Amazonian floodplain lakes. At low water, the influence of parent rivers on floodplain lakes is substantially reduced, and some lakes become totally isolated from their associated rivers (Thomaz et al., 2007). The degree of dissociation with the parent river is, however, fairly variable among lakes, which results from differences in local inputs (Forsberg et al., 1988). Therefore, there are two factors that act simultaneously during low waters: (1) the backwater effect that tends to keep water level higher than expected by discharge, ultimately making lower basin less confined than upper basin ones; and (2) the rate of local inputs of water and associated chemical compounds.

The relative importance of local inputs depends on the hydraulic loading rate from the local drainage basin, which in turn depends on the drainage basin area to lake area ratio (BA:LA; Forsberg et al., 1988). Generally, lakes with a low BA:LA display a mixture of river and local water by the end of the low water period, whereas lakes with a high BA:LA are primarily characterized by the presence of local water. In Amazonian floodplain lakes, the BA:LA ratio can vary by up to two orders of magnitude from one lake to another (Forsberg et al., 1988) – and, in general, higher BA:LA leads to decreased nutrient availability because local water derived from forest runoff is usually less nutrient-enriched. The distance to the Amazon River (i.e., a proxy to the intensity of the backwater effect) had a significantly positive, but low explicability on bacteria and viruses. This low explicability is expected if one considers that a wide range of geological, hydrological, and environmental factors controls planktonic food webs and lake water chemistry. Therefore, we suggest that the BA:LA ratio is likely an important additional factor governing bacteria and viruses in the Trombetas floodplain lakes, as this ratio influences the availability of nutrients and DOM, as well as mixing and dilution of water.

### RELATIONSHIPS OF VIRUSES WITH BACTERIA AND PHYTOPLANKTON

A strong virus–bacterium relationship plus a lack of relationship between virus and phytoplankton indicate that most viruses are bacteriophages (i.e., infect bacteria). The predominance of bacteriophages suggests that VAs increase with distance to the Amazon River because of increased BAs, as viral infection depends directly on the number of host cells (Brussaard, 2004). The predominance of bacteriophages in the lakes studied here is in line with the only existing report of virus–bacterium relationship in Amazonian aquatic ecosystems (Barros et al., 2010), which shows a strong correlation between bacterial and VAs, constant VBR and predominance of bacteriophages. Since the encounter between virus and host cell is mediated by random drift in the water column (Brussaard, 2004), it is expected that bacteriophages predominate in the oligotrophic lakes studied here. Indeed, VAs are usually more strongly correlated to BAs than to phytoplankton in surface waters (Cochlan et al., 1993; Fuhrman, 1999). Also, the relative importance of bacteria over phytoplankton increases in oligotrophic lakes (Cotner and Biddanda, 2002), which reinforces that a strong correlation between viral and BAs is likely to occur in clear-water Amazonian floodplain lakes. Finally, a lack of relationship between phytoplankton and bacteria is consistent with the fact that a low proportion of phytoplankton carbon is transformed into bacterial biomass in the tropics (Roland et al., 2010), probably because most of carbon utilized by tropical aquatic bacteria is potentially used to maintain their high respiration rates (Amado et al., 2013).

The VBR is highly variable in world lakes, with reported ratios ranging from 0.4 to over 100 (Maranger and Bird, 1995; Anesio et al., 2004; Clasen et al., 2008). In tropical systems, reported VBRs range from 4 to 22 (Peduzzi and Schiemer, 2004; Bettarel et al., 2006; Araújo and Godinho, 2009). Therefore, the VBRs of the lakes surveyed here are low (2.5-9.1; average = 4.7), fitting the lower range of values reported for tropical lakes. Nevertheless, our VBR is similar to the only existing description for clear-water floodplain lakes (4.3-6.1; Barros et al., 2010). In the Trombetas River main channel, we observed the highest VBR among all systems, as BA was within the lower range and VA was within the middle range of our dataset. A high abundance of viruses relative to bacteria in the Trombetas River is probably because the more turbulent riverine waters may facilitate the random encounter between viral and bacterial host cells. Finally, both bacterial and VAs were within the middle range of worldwide data (e.g., Maranger and Bird, 1995; Anesio et al., 2004; Bettarel et al., 2006; Clasen et al., 2008), which is in agreement with previous studies in Amazonian lakes (Anesio et al., 1997; Amado et al., 2006; Barros et al., 2010).

### HYDRODYNAMICS AND THE PLANKTONIC VIRAL LOOP

**FIGURE 5:**
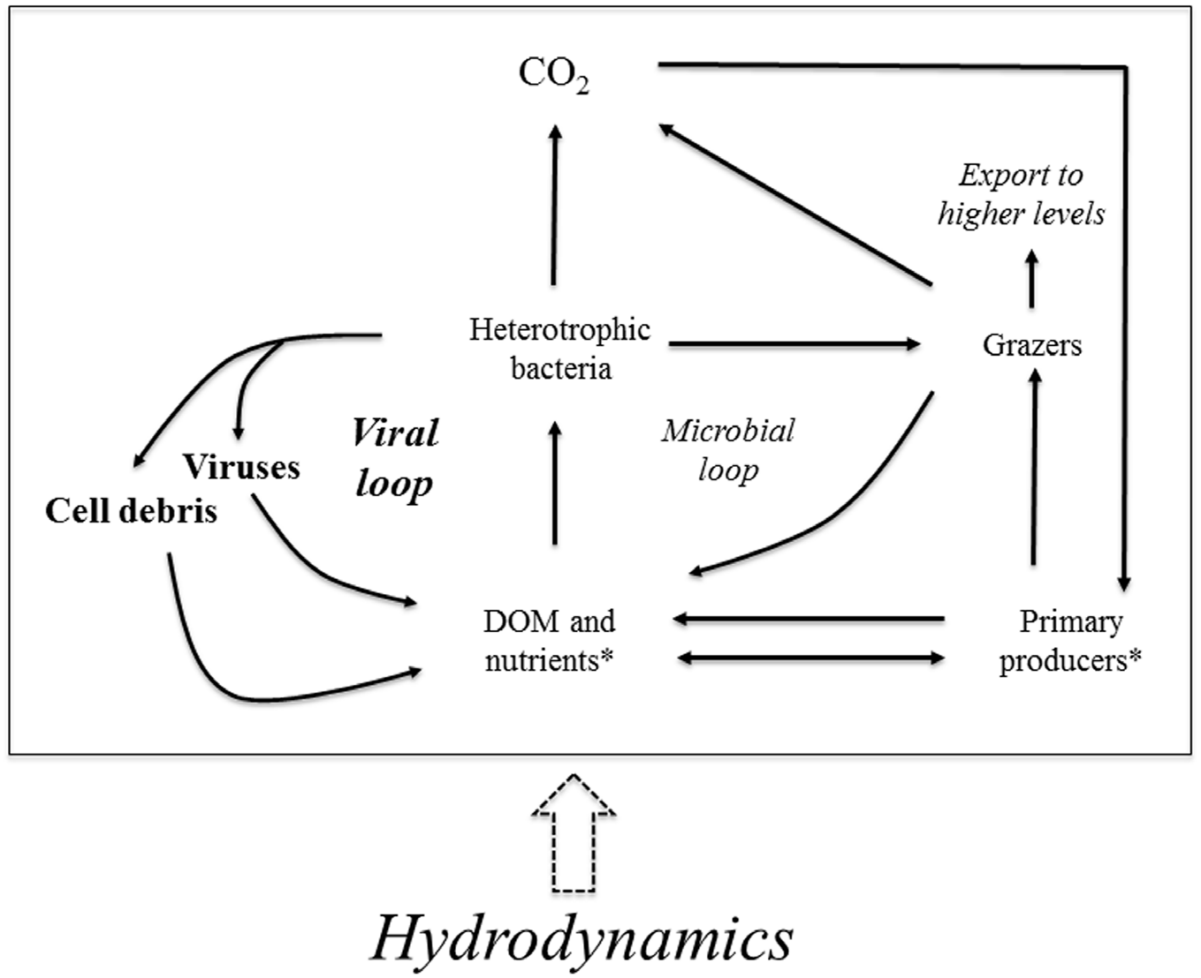
**Schematic diagram representing the viral loop, included within the microbial food web, in Amazonian lakes, emphasizing the importance of hydrodynamics.** There is a semi-closed loop connecting bacteria, viruses, and dissolved organic matter (DOM). Primary producers and grazers are the two external suppliers of DOM to the loop. In Amazonian lakes, however, hydrodynamics is also a pivotal forcing influencing the loop, as it regulates the availability and quality of DOM. Additionally, hydrodynamics promotes water dilution and mixing that change the abundance of the different microbial compartments of the scheme. Modified from Fuhrman (1999). *there are two different arrows connecting “primary producers” with “DOM and nutrients” because primary producers uptake and release nutrients, but they only release DOM.

The viral loop is a semi-closed loop connecting bacteria, viruses, and organic matter. It was initially idealized for marine systems (Fuhrman, 1999), in which the main external suppliers of DOM to the loop are grazers and primary producers. However, we propose that, in Amazonian floodplain lakes, there is a very relevant force that regulates the loop: hydrodynamics (**Figure 5**). A previous study showed that the flood pulse influences bacteria and viruses, with decreased abundances of both communities during floods (Barros et al., 2010); here, we show that viral and BAs increase in lakes less affected by the Amazon River backwater effect. Hydrodynamics also modulates viral communities in macrotidal estuaries, with VAs decreasing seaward because of dilution of viruses entering the estuary from the river (Auguet et al., 2005). Hence, our study builds on previous findings, underscoring the central role of hydrodynamics in shaping the viral loop. The action of hydrodynamics is not only through the regulation of the availability of DOM and nutrients, but also directly through water dilution and mixing of the microbial compartments. The schematic diagram that we propose underpins the role of viruses in the biogeochemistry of Amazonian aquatic ecosystems. Planktonic models indicate that bacterial respiration increases substantially in the presence of viral infection (Fuhrman, 1999). Hence, on the one hand, viral infection may contribute to CO_2_ production – which is large in Amazonian surface waters (Richey et al., 2002; Abril et al., 2014), but on the other hand, this can possibly be counteracted by nutrient regeneration through viral lysis that ultimately favors the growth of primary producers (Shelford et al., 2012).

## CONCLUSION

Although we do not have data for the high water period, existing literature data allow us to make some inferences. Data from 10 floodplain lakes in the Trombetas River basin indicate that there is a higher coefficient of variation for several limnological parameters – including water transparency and DOC – during low waters (Thomaz et al., 2007). This suggests that the lakes are more similar among themselves and with Trombetas River during floods, when they are connected. Thus, the inter-lake dissimilarity of bacterial and VAs that we found during low water is probably less significant during high water due to the increased connectivity. In summary, we found a latitudinal gradient in the characteristics of the floodplain lakes analyzed here. We attribute this spatiality to the backwater effect of the Amazon River on the Trombetas River, which tends to increase the ratio of river to local water in lower basin lakes. DOC enrichment, CO_2_ supersaturation, water transparency, VAs and BAs significantly increase as distance to the Amazon River increases.

## Conflict of Interest Statement

The authors declare that the research was conducted in the absence of any commercial or financial relationships that could be construed as a potential conflict of interest.
